# Clinical prediction models for acute kidney injury

**DOI:** 10.5935/0103-507X.20200018

**Published:** 2020

**Authors:** Chao-Yuan Huang, Fabian Güiza Grandas, Marine Flechet, Geert Meyfroidt

**Affiliations:** 1 Laboratory of Intensive Care Medicine, Academic Department of Cellular and Molecular Medicine, Katholieke Universiteit Leuven - Leuven, Belgium.; 2 Department of Intensive Care Medicine, University Hospitals Leuven - Leuven, Belgium.

**Keywords:** Acute kidney injury, Models, theoretical, Intensive care units, Lesão renal aguda, Modelos teóricos, Unidades de terapia intensiva

## Abstract

**Objective:**

To report on the currently available prediction models for the development of acute kidney injury in heterogeneous adult intensive care units.

**Methods:**

A systematic review of clinical prediction models for acute kidney injury in adult intensive care unit populations was carried out. PubMed^®^ was searched for publications reporting on the development of a novel prediction model, validation of an established model, or impact of an existing prediction model for early acute kidney injury diagnosis in intensive care units.

**Results:**

We screened 583 potentially relevant articles. Among the 32 remaining articles in the first selection, only 5 met the inclusion criteria. The nonstandardized adaptations that were made to define baseline serum creatinine when the preadmission value was missing led to heterogeneous definitions of the outcome. Commonly included predictors were sepsis, age, and serum creatinine level. The final models included between 5 and 19 risk factors. The areas under the Receiver Operating Characteristic curves to predict acute kidney injury development in the internal validation cohorts ranged from 0.78 to 0.88. Only two studies were externally validated.

**Conclusion:**

Clinical prediction models for acute kidney injury can help in applying more timely preventive interventions to the right patients. However, in intensive care unit populations, few models have been externally validated. In addition, heterogeneous definitions for acute kidney injury and evaluation criteria and the lack of impact analysis hamper a thorough comparison of existing models. Future research is needed to validate the established models and to analyze their clinical impact before they can be applied in clinical practice.

## Abstract (pt)

**Objetivo:**

Relatar os modelos atualmente disponíveis de predição para o desenvolvimento de lesão renal aguda em unidades de terapia para adultos heterogêneas.

**Métodos:**

Foi realizada revisão sistemática dos modelos de predição de lesão renal aguda em unidades de terapia intensiva para pacientes adultos. Fizemos busca na base PubMed^®^ quanto a publicações que relatassem desenvolvimento de um novo modelo de predição, validação de um modelo estabelecido, ou impacto de um modelo de predição existente para diagnóstico precoce de lesão renal aguda em unidades de terapia intensiva.

**Resultados:**

Triamos 583 artigos potencialmente relevantes. Dentre os 32 artigos que restaram após a primeira seleção, apenas 5 cumpriram os critérios para inclusão. As adaptações não padronizadas feitas para definir o valor inicial de creatinina sérica, quando o valor desse exame antes da admissão estava faltando, levaram a definições heterogêneas do resultado. Preditores comumente incluídos foram sepse, idade e nível de creatinina sérica. Os modelos finais incluíram entre 5 e 19 fatores de risco. As áreas sob a curva Característica de Operação do Receptor para predição do desenvolvimento de lesão renal aguda nas coortes de validação interna variaram entre 0,78 e 0,88. Apenas dois estudos tiveram validação externa.

**Conclusão:**

Os modelos de predição clínica de lesão renal aguda podem ajudar na aplicação em tempo apropriado de intervenções preventivas para os pacientes adequados. Contudo, poucos modelos foram externamente validados para as populações da unidade de terapia intensiva. Além disto, definições heterogêneas para lesão renal aguda e critérios de avaliação, e a falta de análise do impacto comprometem uma comparação abrangente dos modelos existentes. São necessárias novas pesquisas para validar os modelos estabelecidos e analisar seu impacto clínico, antes que estes possam ser aplicados na prática clínica.

## INTRODUCTION

Critically ill patients need specific support to preserve the function of their vital organs, for which they are treated in intensive care units (ICU).^(1,2)^ Predicting the future health state of these patients is crucial, and clinicians try to foresee changes in their clinical situation as early as possible to adjust the treatments, prevent organ failure at an early stage and avoid unfavorable outcomes. Acute kidney injury (AKI) is one of the most prevalent organ failures in critically ill patients, affecting approximately 40% of ICU admissions.^(3-6)^Acute kidney injury is defined as a rapid decline in renal excretory function and is classified into three stages according to the degree of increase in serum creatinine (SCr) and/or the decline in urine output (UO).^(7)^However, both criteria are late and nonspecific signs of actual underlying kidney damage. For the early detection of AKI, several plasma or serum and urinary biomarkers have been identified and are available through diagnostic tests,^(8-15)^even though it is still unclear which patient populations would benefit from biomarker testing, what the timing of said testing should be, or what the therapeutic consequences of a positive test could be in the absence of a specific therapy for AKI. In addition, these biomarkers carry a certain financial cost.

The ICU is a data-rich environment. The typical critically ill patient, with continuous monitoring of vital signs, therapeutic devices, radiological imaging and serial laboratory measurements, yields large amounts of data. Electronic health record implementation is increasing worldwide,^(16)^making these data available for analysis, and as such, the ICU is an ideal environment to transform these data into valuable information for prediction. These large and complex electronic health record databases present a challenge for traditional statistical techniques, however. Automatic learning algorithms, also known as machine learning, could offer a better understanding of the complex variability and interactions among high-dimensional variables.^(17)^

The goal of the present review is to investigate which internally validated clinical prediction models for early AKI diagnosis in the heterogeneous adult ICU are currently available.

## METHODS

### Search strategy

PubMed^®^ was searched for review articles and full reports of retrospective and prospective studies that were published in English with full text availability from January 1^st^, 2012 to June 5^th^, 2019. Due to the lack of a unified definition for AKI prior to the Kidney Disease: Improving Global Outcomes (KDIGO) AKI criteria proposed in 2012,^(7)^we only investigated studies published over the past 7 years.

A combination of three search terms was used: “intensive care unit”, “acute kidney injury”, and “prediction”. On the basis of a previous review,^(18)^we adapted these words into Medical Subject Heading (MeSH) terms and keywords in a title and abstract search (Table 1). We manually examined all the included studies. Potentially relevant articles identified by other sources and references of the retrieved literature were also included in the examination.

**Table 1:** Search strategy

MeSH term		Title/abstract
Acute kidney injury, renal replacement therapy, renal dialysis, renal insufficiency	OR	acute kidney injury, renal insufficiency*, acute renal failure, renal replacement therapy, dialysis*, peritoneal dialysis*, hemodialysis*, hemodiafiltration
		AND
Decision support techniques	OR	predict* model, predict* rule, predict* score, prognosis* model, nomogram*, decision rule, risk model*, risk algorithm*, validation, risk index, risk predict*, clinical model
		AND
Intensive care units, critical illness	OR	intensive care unit*, critical ill*

Source: adapted from Wilson T, Quan S, Cheema K, Zarnke K, Quinn R, de Koning L, et al. Risk prediction models for acute kidney injury following major noncardiac surgery: systematic review. Nephrol Dial Transplant. 2016;31(2):231-40. ^(18)^ MeSH - Medical Subject Headings.

### Inclusion criteria

Reviewers first screened all abstracts and titles of the articles that were retrieved by the abovementioned strategy. Articles recognized as relevant were further read in full if they developed a novel prediction model for early AKI diagnosis in the ICU, validated an established model for early AKI diagnosis in the ICU, or appraised the impact of an existing prediction model for early AKI diagnosis in the ICU. A prediction model for early AKI diagnosis was defined as a model using more than one risk factor to estimate the probability of AKI development.

### Exclusion criteria

Studies were excluded if they were performed on patients outside the ICU setting; were performed in pediatric patients or infants; did not validate the models formally, internally or externally;^(19)^focused exclusively on biomarkers and did not contain a clinical prediction model for AKI; lacked formal performance measures (at a minimum, the area under the receiver operating characteristics curve - AUROC/c-statistic should have been reported).

### Data extraction

A data extraction form was used in accordance with previous reviews and guidelines.^(18-20)^ Extracted items included study type (e.g., prospective, retrospective, case-control or cohort studies), population (e.g., cardiac, septic, or surgical patients), modeling method (e.g., multivariate logistic regression), AKI definition (e.g., Acute Kidney Injury Network - AKIN - or KDIGO), internal validation method (e.g., random split, cross-validation, or bootstrap), number of patients, type and number of predictors, and model performance (e.g., discrimination, calibration, or decision curve analysis). External validation results were reported when available.

### Model performance

The evaluation of model performance concentrated on model discrimination, calibration, and decision curve analysis. Discrimination refers to the ability of a model to discriminate patients with a specific symptom from those without it. One common way to report model discrimination is to visualize the ROC curve and report the AUROC.^(21)^Calibration refers to the agreement between predicted probabilities and observed outcome proportions in the population.^(22)^ Common methods used to evaluate model calibration include calibration in the large, calibration slope, and the Hosmer-Lemeshow test.^(23)^A well-calibrated model should have an Hosmer-Lemeshow test p-value larger than 0.05, a calibration slope close to one, and a calibration in the large close to zero. It is also encouraged to plot the observed outcome proportions versus predicted probabilities along with an indication of the calibration slope.^(19)^ Decision curve analysis refers to the quantification of the net benefit of the prediction model across a range of different possible classification thresholds.^(24)^ For a specific threshold, the prediction model with the highest net benefit will be the most clinically useful.

## RESULTS

**Figure 1:**
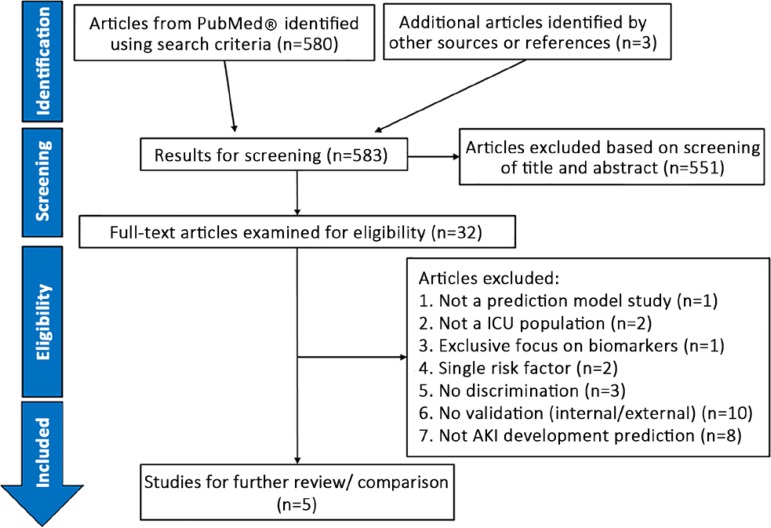
Preferred Reporting Items for Systematic Reviews and Meta-Analyses (PRISMA) four-phase flow diagram.^(40)^ ICU - intensive care unit; AKI - acute kidney injury.

We identified 583 potentially relevant articles, of which 551 were excluded based on screening titles and abstracts. We further read the 32 remaining studies in full, and five were retained for detailed comparison as they satisfied the inclusion criteria (Figure 1). A comparative summary of the clinical prediction models for AKI in the ICU is shown in table 2.

**Table 2:** Comparison of clinical prediction models for early acute kidney injury diagnosis in adult intensive care units

Characteristic	Characteristic subgroup	Malhotra et al.^(25)^	Flechet et al.*^(26)^	Deng et al.^†^^(28)^	Chiofolo et al.^(29)^	Zimmerman et al.^‡^^(30)^
Year of publication		2017	2017	2017	2019	2019
Sample size	Development cohort	573	2,123	1,084	4,572	19,160
	Internal validation cohort	144	2,123	1,084	1,958	4,790
	External validation cohort	1,300	2,367 (independent split set of the original data)	Not applicable	Not applicable	Not applicable
Study type	Prospective or retrospective	Prospective	Retrospective	Prospective	Retrospective	Retrospective
	Single-center or multicenter	Multicenter	Multicenter	Multicenter	Single-center	Single-center
Patient population		Adult patients admitted to ICU without known AKI at enrollment	Adult patients admitted to ICU without a history of ESRD and a baseline SCr ≥ 4mg/dL	Adult patients admitted to ICU	Adult patients admitted to ICU without ESRD and history of AKI	Adult patients admitted to ICU without CKD and AKI on admission
AKI definition	Criteria	KDIGO SCr criteria	KDIGO SCr criteria	KDIGO SCr and/or UO criteria	AKIN SCr and/or UO criteria	KDIGO SCr criteria
	Baseline SCr	SCr measurements 7 to 365 days before ICU admission	SCr measurements 1 week to 3 months before	SCr measurements 3 to > 365 days before ICU admission	SCr measurements 180 days before ICU admission	Day1 minimum SCr
	Imputed baseline SCr	SCr at ICU admission	MDRD back calculation	SCr at ICU admission, or follow-up up to 365 days	MDRD back calculation	Not applicable since no patients lacked baseline SCr
Incidence of outcome (%)	Development cohort	22	27.7	30.1	30	16.5
	Internal validation cohort	24	27.7	30.1	30	16.5
	External validation cohort	45	29.2 (independent split set of the original data)	Not applicable	Not applicable	Not applicable
Missing preadmission SCr values (%)	Development cohort	24	22.8	36.0	Not reported	Not reported
	Internal validation cohort	24	22.8	36.0	Not reported	Not reported
	External validation cohort	30	22.9 (independent split set of the original data)	Not applicable	Not applicable	Not applicable
Timeframe	Collection of risk predictors	Within 48 hours after ICU admission	Within 1 day after ICU admission	Within 1 day after ICU admission	Up to 1 day before the time of prediction	Within 1 day after ICU admission
	Prediction	Within 1 week after study enrollment	Within 1 week after ICU admission	Within 1 week after ICU enrollment	Every 15 minutes after ICU admission	Within 72 hours after ICU admission
Number of risk factors for any AKI		10	12	5	19	16
Techniques	Variable selection	Stepwise forward elimination	Backward elimination method	Stepwise method	Stepwise regression algorithm	Backward elimination method
	Modeling algorithm	MLR	Random forest	ULR and MLR	Random forest	MLR
	Internal validation	5-fold cross-validation	Bootstrapping	Bootstrapping	Random split	5-fold cross-validation
	External validation	Independent prospective cohort	Independent split set of the original data	Not applicable	Not applicable	Not applicable
Discrimination (AUROC)	Development cohort	Not reported	0.86 (0.86 - 0.86)	0.821 (0.792 - 0.850)	0.949 (0.943 - 0.954)	Not reported
	Internal validation cohort	0.792 (0.697 - 0.887)	0.86 (0.86 - 0.86)	0.821 (0.792 - 0.850)	0.882 (0.867 - 0.897)	0.78
	External validation cohort	0.81 (0.78 - 0.83)	0.81 (0.81 - 0.81)	Not applicable	Not applicable	Not applicable
Calibration	Development cohort	Not reported	Calibration slope: 0.87 (0.87 - 0.88),	Not applicable	H-L test p-value:	Not applicable
			Calibration in the large: -0.00, and calibration curve		0.3, and calibration curve	
	Internal validation cohort	Hosmer-Lemeshow test p-value: 0.293, and calibration curve	Calibration slope: 0.87 (0.87 - 0.88),	Not applicable	Hosmer-Lemeshow test p-value: 0.3, and calibration curve	Not applicable
			calibration in the large: -0.00 (-0.01 - 0.00), and calibration curve			
	External validation cohort	Calibration curve	Calibration slope: 0.78 (0.78 - 0.79), calibration in the large: -0.01 (-0.01 – -0.01), and calibration curve	Not applicable	Not applicable	Not applicable

ICU - intensive care unit; AKI - acute kidney injury; ESRD - end-stage renal disease; SCr - serum creatinine; CKD - chronic kidney disease; KDIGO - Kidney Disease: Improving Global Outcomes; MDRD - Modification of Diet in Renal Disease; UO - urine output; AKIN - Acute Kidney Injury Network; MLR - multivariate logistic regression; ULR - univariate logistic regression; AUROC - area under the receiver operating characteristics.

* Data from Flechet et al. are only reported for the prediction model for any AKI on the first day;

† data from Deng et al. are only reported for the prediction model for any AKI;

‡ data from Zimmerman et al. are only reported for the multivariate logistic regression model derived with the backward elimination method.

Malhotra et al.^(25)^conducted a prospective multicenter study in which they developed and internally as well as externally validated a risk score for early AKI diagnosis in ICU patients. First, predictor candidates within 48 hours after ICU enrollment were selected based on previous studies. Second, stepwise forward selection was used for feature selection. Third, the prediction model was built on a development cohort using multivariate logistic regression methodology with 5-fold cross-validation to predict the occurrence of all stages of AKI (KDIGO SCr criterion stages 1, 2, and 3) in the first week of ICU stay. Afterwards, they converted the coefficient-based regression prediction model to a point-based risk score ranging from zero to 21. They examined the generalizability of the risk score model by internally validating the model on a test cohort and externally validating it on an independent cohort. The AUROC for internal and external validation were 0.79 (95% of confidence interval - 95%CI 0.70 - 0.89) and 0.81 (95%CI 0.78 - 0.83), respectively. Calibration was reported with an Hosmer-Lemeshow test p-value of 0.293 for internal validation and with calibration plots for the internal and external validation cohorts. Since the Hosmer-Lemeshow test p-value was larger than 0.05 and there was agreement between the observed outcome proportions and predicted probabilities in the calibration curves, the prediction model was considered well calibrated.

AKI Predictor,^(26)^an online prognostic calculator for early AKI diagnosis in the ICU, was created based on the database from a large multicenter randomized control trial.^(27)^ Four prediction models were developed based on clinical information available at different time points: before ICU admission (baseline model), upon ICU admission (admission model), on the first day of ICU admission (day1 model), and 24 hours after ICU admission (day1+ model). First, candidate predictors were considered on the basis of the literature, expert opinion, and availability in the dataset. Second, feature selection was performed by using bootstrapped backward elimination analysis. Third, models were developed on a development cohort using a random forest algorithm to predict the occurrence of all stages of AKI (KDIGO SCr criterion stages 1, 2, and 3; AKI-123) and the more severe stages (KDIGO SCr criterion stages 2 and 3; AKI-23) separately in the first week of ICU stay. Fourth, the performance of the clinical model was internally validated by bootstrapping. Subsequently, models were validated on an unseen independent validation cohort, where the prognostic performance was compared against neutrophil gelatinase-associated lipocalin (NGAL) measurements obtained at ICU admission. The AUROC of the day 1 model for AKI-123 for the development and validation cohorts were 0.86 (95%CI 0.86 - 0.86) and 0.81 (95%CI 0.81 - 0.81), respectively. The AUROC of NGAL for AKI-123 for the validation cohort was 0.67 (95%CI 0.67 - 0.67). The calibration slopes of the day 1 model for AKI-123 for the development and validation cohorts were 0.87 (95%CI 0.87 - 0.88) and 0.78 (95%CI 0.78 - 0.79), respectively. The decision curves of the day 1 model for AKI-123 showed clinical usefulness for classification thresholds widely ranging from 4.2% to 72.4% in the validation cohort.

Deng et al.^(28)^carried out a prospective multicenter observational study of the efficacy of three individual biomarkers (serum cystatin C - sCysC -, urinary N-acetyl-β-D-glucosaminidase - uNAG -, and the urinary albumin/creatinine ratio - uACR) and their combinations on the early diagnosis of AKI in ICU patients. First, to predict the occurrence of all stages of AKI (KDIGO SCr criterion stages 1, 2, and 3; any AKI) and the more severe stages (KDIGO SCr criterion stages 2 and 3; severe AKI) separately in the first week of ICU stay of the patient, the authors looked into the performance of the three biomarkers separately. Second, to improve the performance of these biomarkers in AKI detection, they created three models consisting of different biomarker combinations and examined their performance. Third, the clinical variables that were shown to be significantly different between patients with and without AKI (two-sided p-value less than 0.1) in univariate analysis within 1 day after ICU admission were regarded as candidates in the final model. Fourth, a stepwise selection was utilized to select variables used in the final prediction models. Fifth, they built prediction models for the early diagnosis of AKI with multivariate logistic regression, investigated the contribution of biomarkers to the clinical prediction models, and internally validated the performance by bootstrapping. The AUROC of the multivariate models of sCysC and uNAG for any AKI and severe AKI were 0.756 (95%CI 0.723 - 0.789) and 0.863 (95%CI 0.827 - 0.900), respectively. The AUROC of the prediction models for any AKI and severe AKI were 0.821 (95%CI 0.792 - 0.850) and 0.908 (95%CI 0.881 - 0.934), respectively. The AUROC of the prediction models combined with sCysC and uNAG for any AKI and severe AKI were 0.836 (95%CI 0.808 - 0.864) and 0.918 (95%CI 0.893 - 0.944), respectively.

Chiofolo et al.^(29)^ performed a single-center retrospective study on a heterogeneous cohort of adults admitted to ICUs, with the aim of developing and validating a prediction model for AKI development of any stage (AKIN SCr and/or UO criteria without inclusion of dialysis rules). First, predictors up to 24 hours before the time of prediction with AUROC larger than 0.5 in the univariate analysis were selected. Second, selected predictors were further chosen based on their AUROC and clinical judgment. Third, a stepwise regression algorithm was conducted to confirm the predictor selection. Fourth, random forest models were built with different feature combinations. Fifth, the final model was selected based on the AUROC and early AKI detection percentage and internally validated in a cohort that was randomly split from the original data. Finally, by adjusting different classification thresholds, the prediction model could be used to detect any stage of AKI or only moderate-severe AKI. To continuously monitor ICU patients, the prediction model was designed to output the AKI development probability every 15 minutes from ICU admission to discharge. The AUROCs in the development and validation cohorts were 0.949 (95%CI 0.943 - 0.954) and 0.882 (95%CI 0.867 - 0.897), respectively. The well-calibrated performance was confirmed by an Hosmer-Lemeshow test p-value of 0.3. In addition, based on the highest net benefit of the model across a wide range of classification thresholds (approximately from 5% to 95%) in the decision curve analysis, the clinical usefulness of the model was verified.

Zimmerman et al.^(30)^ reported a retrospective single-center study in which they developed and validated prediction models for AKI development of any stage (KDIGO SCr criteria). First, demographics, clinical data and laboratory test measurements within 1 day after ICU admission that were identified as AKI risk factors in previous studies were selected. Second, variables missing more than 20% of their values were removed, and the missing values of the remaining variables were imputed by using multivariate imputation by chained equations (MICE). Third, variables that were not significantly associated with the outcome in the univariate analysis (p-value > 0.05) were excluded. Fourth, a backward elimination method was applied to variables significantly associated with the outcome (p-value £ 0.05). Fifth, prediction models for AKI development within 72 hours after ICU admission were developed by using multivariate logistic regression, a random forest method, and a multilayer perceptron based on the selected variables. Sixth, the models were internally validated by ten runs of 5-fold cross-validation, and measurements were averaged over the ten runs for models with and without use of the backward elimination method. The averaged AUROC in the internal validation cohort for multivariate logistic regression, the random forest method, and the multilayer perceptron models with backward selection were 0.780, 0.772, and 0.792, while the averaged AUROC without backward elimination method were 0.783, 0.779, and 0.796, respectively.

## DISCUSSION

In this systematic review, we identified five studies in which clinical prediction models for the early diagnosis of AKI were built and validated in heterogeneous cohorts of ICU patients. One study focused on the assessment of performance with and without the inclusion of biomarkers in clinical prediction models.^(28)^AKI was defined using the KDIGO criteria in four studies,^(25,26,28,30)^ among which three studies did not include the UO criteria,^(25,26,30)^ and one study used the AKIN criteria without inclusion of dialysis rules.^(29)^Definitions of baseline SCr and methods to handle missing pre-ICU values were heterogeneous, hindering model comparison. Three models reported calibration measures.^(25,26,29)^ Only two models were validated externally.^(25,26)^The number of independent predictors in the final models ranged from 5 to 19, but no single predictor was used consistently in all studies. To the best of our knowledge, to date there has been no impact analysis of these prediction models in practical clinical settings.

### AKI definition and severity

Although the discussed models were mostly based on the latest unified KDIGO AKI criteria,^(25,26,28,30)^we found that the AKI definitions had been adapted when the necessary data were not available. First, three studies defined AKI using the KDIGO SCr criterion without including UO.^(25,26,30)^This definition is understandable in view of the difficulty of measuring and recording UO every hour, which requires manual data entry. Nevertheless, not using UO, which is an important component of the AKI definition, is suboptimal since the change in UO could be a more sensitive marker of renal dysfunction compared to SCr.^(31)^A second and perhaps even more crucial issue was the lack of a standardized method to define the baseline SCr concentration, which largely influenced the SCr change-based definition and classification of AKI. To determine the baseline SCr, one study used the day 1 minimum creatinine level,^(30)^while the others used preadmission SCr values. Regarding missing data in the preadmission values, two studies used admission SCr.^(25,28)^and the other two studies estimated the baseline SCr by back calculation from the Modification of Diet in Renal Disease (MDRD) formula with an assumed normal glomerular filtration rate.^(26,29)^However, none of these estimations is without problems. Studies have shown that MDRD back calculation may overestimate the incidence of AKI and admission SCr underestimates it.^(32)^

With respect to the severity of predictive AKI outcome, two studies investigated all stages of AKI (stage 1, 2, or 3 AKI),^(25,30)^and the others looked into both moderate to severe AKI (stage 2 or 3 AKI) and all stages of AKI.^(26,28,29)^The stronger association between predictors and outcome for higher severity AKI^(33)^could explain the more robust performance of the models built for moderate to severe AKI.

### Validation, calibration, and decision curve analysis

To prevent overly optimistic claims to the performance, one study utilized random split,^(29)^two used cross-validation,^(25,30)^and the others employed bootstrapping^(26,28)^ for internal validation. It is recommended to use bootstrapping to estimate internal validity, as it provides stable estimates and reduces bias.^(34,35)^Two studies properly externally validated their models,^(25,26)^while the others did not perform external validation.^(28-30)^ External validation is a necessary step to assess the generalizability of a model in a previously unseen setting. The more the models are externally validated in diverse settings, the more their generalizability can be trusted.

Although all five studies reported fair discrimination, two studies did not examine performance with respect to calibration.^(28,30)^A prediction model is of no practical use if it achieves high discrimination but poor calibration because calibration demonstrates whether the model applies to the population examined. Furthermore, in three studies that included a calibration measure, one study used calibration in the large and the calibration slope with the calibration curve,^(26)^and the others used the Hosmer-Lemeshow test with the calibration curve.^(25,29)^ Notably, even though the Hosmer-Lemeshow test is widely used, it is sensitive to sample size and incidence of the predicted outcome.^(36)^ Therefore, caution should be exercised when assessing model calibration by the Hosmer-Lemeshow test.

Additionally, two of the five studies further conducted decision curve analysis.^(26,29)^Both studies showed the clinical usefulness of their models for a wide range of classification thresholds. Decision curve analysis is recommended not only because it allows the choosing of a model with the highest net benefit but also because it provides a range of clinically useful classification thresholds.

### Study design, comparison with biomarkers, and commonly included risk predictors

Two studies were prospective,^(25,28)^ three studies were multicentered with sample sizes greater than 500 patients,^(25,26,28)^ and all of the studies were based on databases of mixed critically ill patients admitted to the ICU. The large sample sizes in the studies provided crucial insights into the development of prediction models for the early diagnosis of AKI in ICU populations, which complemented reviews in general hospital populations,^(37)^ patients after liver transplantation,^(38)^ patients after cardiac surgery,^(39)^ and major non-cardiac surgical patients.^(18)^ Data from multicentered studies have greater heterogeneity, which leads to models with lower chances of overfitting. The incidence of AKI ranged from 16.5% to 30.1%.

Although it met the inclusion criteria and thus included at least one prediction model, one study was designed specifically to examine the predictive performance of biomarkers instead of clinical prediction models.^(28)^ To investigate early AKI diagnosis in ICU populations, the authors investigated three biomarkers (sCysC, uNAG, uACR) and their combinations.^(28)^ In contrast, the remaining four studies focused on prediction models,^(25,26,29,30)^among which one made a comparison between prediction models with and without inclusion of a biomarker (NGAL).^(26)^ A comparison between biomarkers, prediction models, and combined models for early AKI diagnosis in adult ICUs is presented in table 3. In the two studies that compared biomarkers and prediction models,^(26,28)^ the prediction models outperformed biomarkers with regard to discrimination. It is also noteworthy that after combining the biomarkers with the clinical prediction models, improved discrimination was reported in both studies, but whether the small increase in discrimination after biomarker inclusion compensates for the added costs of performing a biomarker test is debatable.

**Table 3:** Comparison between biomarkers, prediction models, and combined models for early acute kidney injury diagnosis in adult intensive care units

Characteristic	Characteristic sub-groups	Malhotra et al.^(25)^	Flechet et al.*^(26)^	Deng et al.^†^^(28)^	Chiofolo et al.^(29)^	Zimmerman et al.^‡^^(30)^
Biomarkers used for comparison		Not applicable	sNGAL	sCysC and uNAG	Not applicable	Not applicable
Discrimination of biomarkers (AUROC)		Not applicable	For NGAL in validation NGAL cohort: 0.74 (0.74 - 0.74)	0.756 (0.723 - 0.789)	Not applicable	Not applicable
Discrimination of prediction models (AUROC)	Development cohort	Not reported	0.86 (0.86 - 0.86)	0.821 (0.792 - 0.850)	0.949 (0.943 - 0.954)	Not reported
	Internal validation cohort	0.792 (0.697 - 0.887)	0.86 (0.86 - 0.86)	0.821 (0.792 - 0.850)	0.882 (0.867 - 0.897)	0.78
	External validation cohort	0.81 (0.78 - 0.83)	0.81 (0.81 - 0.81)	Not applicable	Not applicable	Not applicable
Discrimination of prediction models with biomarkers (AUROC)		Not applicable	For combined model in validation NGAL cohort: 0.80 (0.80 - 0.80)	0.836 (0.808 - 0.864)	Not applicable	Not applicable

sNGAL - serum neutrophil gelatinase-associated lipocalin; sCysC - serum cystatin C; uNAG - urinary N-acetyl-β-D-glucosaminidase; AUROC - area under the receiver operating characteristics; NGAL - neutrophil gelatinase-associated lipocalin.

* Data from Flechet et al. are only reported for the prediction model for any acute kidney injury on the first day;

† data from Deng et al. are only reported for the prediction model for any acute kidney injury;

‡ data for Zimmerman et al. are only reported for the multivariate logistic regression model derived with backward selection.

Although all models included a variable selection methodology, there was large variability across the different studies in the final variables used. Among the 47 predictive variables identified across the five prediction models, no single variable was used consistently in all studies (Table 4). Nonetheless, sepsis, age, and creatinine level were commonly included predictors (present in more than two studies).

**Table 4:** Risk factors used in clinical prediction models for acute kidney injury in the intensive care unit across studies

Characteristics	Malhotra et al.^(25)^	Flechet et al.*^(26)^	Deng et al.^†^^(28)^	Chiofolo et al.^(29)^	Zimmerman et al.^‡^^(30)^	Total
Demographic variables						
Age		✓		✓	✓	3
Gender			✓		✓	2
Ethnicity					✓	1
Chronic variables						
Baseline SCr		✓				1
Hypertension	✓					1
Diabetes		✓				1
Chronic kidney disease	✓					1
Chronic liver disease	✓		✓			2
Congestive heart failure	✓					1
Atherosclerotic coronary vascular disease	✓					1
Acute variables						
pH value	✓			✓		2
Mechanical ventilation	✓				✓	2
Hemoglobin level	✓				✓	2
Surgical medical category		✓				1
Planned admission		✓	✓			2
Blood glucose upon ICU admission		✓				1
Hemodynamic support upon ICU admission		✓				1
Maximum lactate		✓				1
Bilirubin		✓				1
Creatinine level		✓	✓		✓	3
APACHE II score		✓				1
Nephrotoxic drugs	✓					1
Sepsis	✓	✓	✓			3
Blood urea nitrogen				✓	✓	2
Noninvasive diastolic blood pressure				✓		1
Temperature				✓		1
Noninvasive mean arterial blood pressure				✓		1
Hematocrit				✓		1
Sodium level				✓		1
Potassium level				✓	✓	2
Calcium level					✓	1
Estimated GFR				✓	✓	2
Median urine output at 12 hours				✓		1
Median urine output at 24 hours				✓		1
Shock index based on noninvasive diastolic blood pressure				✓		1
Shock index based on invasive diastolic blood pressure				✓		1
Pulse pressure				✓		1
Delivered tidal volume				✓		1
Partial pressure of arterial oxygen to fraction of inspired oxygen ratio				✓		1
Net fluid balance				✓		1
Cumulative dose of normal saline				✓		1
Systolic blood pressure					✓	1
SpO_2_					✓	1
Bicarbonate level					✓	1
Platelet count					✓	1
Partial thromboplastin time					✓	1
International normalized ratio					✓	1

SCr - serum creatinine; ICU - intensive care unit; APACHE - Acute Physiology and Chronic Health Evaluation; GFR - glomerular filtration rate; SpO_2_: oxygen saturation.

* Data from Flechet et al. are only reported for the prediction model for any acute kidney injury on the first day;

† data from Deng et al. are only reported for the prediction model for any acute kidney injury;

‡ data from Zimmerman et al. are only reported for the multivariate logistic regression model derived with backward selection.

### Limitations

First, the main limitation of this systematic review is the small number of studies included due to the very strict inclusion criteria, which could have prevented us from identifying less formal but novel studies. Second, the protocol of our systematic review was not published in advance in an international database of prospectively registered systematic reviews, such as PROSPERO (https://www.crd.york.ac.uk/prospero). Nevertheless, the literature was searched in a systematic way following an appropriate methodology based recent guidelines.^(40)^ Third, we only used the PubMed^®^ database to search for potentially relevant articles for this systematic review, and as such, we could have missed manuscripts that are available in other databases. Fourth, only studies published in the last seven years were retrieved. This predefined 7-year time window is justified since there were multiple AKI definitions before the publication of the KDIGO AKI criteria in 2012, and we wanted to avoid heterogeneous AKI definitions by only investigating studies completed after the publication of these unified AKI criteria. However, it should still be acknowledged that nonstandardized adaptations that were made to define baseline SCr when the preadmission value was missing still lead to heterogeneous definitions of the outcome. Fifth, no recommendation of one model over the others was given, since only two out of five studies used an external validation cohort, and since no impact analysis was performed. Finally, since we did not obtain individual participant data from these studies, a meta-analysis was not possible.

## CONCLUSION

Several clinical models that can predict acute kidney injury in patients admitted to the intensive care unit have been developed over the past years. Since only two of these models have been validated in an independent cohort, their clinical use remains limited. To make these models robust enough for decision support in clinical practice, three important recommendations need to be followed. First, blinded prospective clinical trials for independent validation need to be set up. Second, these validation studies should use calibration analysis and decision curve analysis, in addition to the more universally reported discrimination analysis, as evaluation criteria. Third, to make the models more generalizable, the use of the KDIGO acute kidney injury criteria and established definitions for baseline serum creatinine is recommended. It is clear that the availability of large high-resolution datasets combined with novel statistical and machine learning tools opens opportunities to develop and validate robust predictive models for acute kidney injury of potential benefit in patient care and risk stratification.

## References

[1] Adhikari NK, Fowler RA, Bhagwanjee S, Rubenfeld GD (2010). Critical care and the global burden of critical illness in adults. Lancet.

[2] Marshall JC, Bosco L, Adhikari NK, Connolly B, Diaz JV, Dorman T (2017). What is an intensive care unit? A report of the task force of the World Federation of Societies of Intensive and Critical Care Medicine. J Crit Care.

[3] Gunst J, Vanhorebeek I, Casaer MP, Hermans G, Wouters PJ, Dubois J (2013). Impact of early parenteral nutrition on metabolism and kidney injury. J Am Soc Nephrol.

[4] Bellomo R, Kellum JA, Ronco C (2012). Acute kidney injury. Lancet.

[5] Joannidis M, Metnitz B, Bauer P, Schusterschitz N, Moreno R, Druml W (2009). Acute kidney injury in critically ill patients classified by AKIN versus RIFLE using the SAPS 3 database. Int Care Med.

[6] Nisula S, Kaukonen KM, Vaara ST, Korhonen AM, Poukkanen M, Karlsson S, Haapio M, Inkinen O, Parviainen I, Suojaranta-Ylinen R, Laurila JJ, Tenhunen J, Reinikainen M, Ala-Kokko T, Ruokonen E, Kuitunen A, Pettilä V, FINNAKI Study Group (2013). Incidence, risk factors and 90-day mortality of patients with acute kidney injury in Finnish intensive care units: The FINNAKI study. Intensive Care Med.

[7] Khwaja A (2012). KDIGO clinical practice guidelines for acute kidney injury. Nephron Clin Pract.

[8] Ostermann M, Joannidis M (2015). Biomarkers for AKI improve clinical practice: no. Intensive Care Med.

[9] Bell M, Larsson A, Venge P, Bellomo R, Mårtensson J (2015). Assessment of cell-cycle arrest biomarkers to predict early and delayed acute kidney injury. Dis Markers.

[10] Prowle JR (2015). Measurement of AKI biomarkers in the ICU: still striving for appropriate clinical indications. Intensive Care Med.

[11] Haase-Fielitz A, Haase M, Devarajan P (2014). Neutrophil gelatinase-associated lipocalin as a biomarker of acute kidney injury: a critical evaluation of current status. Ann Clin Biochem.

[12] Vanmassenhove J, Vanholder R, Nagler E, Van Biesen W (2013). Urinary and serum biomarkers for the diagnosis of acute kidney injury: an in-depth review of the literature. Nephrol Dial Transplant.

[13] Kashani K, Al-Khafaji A, Ardiles T, Artigas A, Bagshaw SM, Bell M (2013). Discovery and validation of cell cycle arrest biomarkers in human acute kidney injury. Crit Care.

[14] Bihorac A, Chawla LS, Shaw AD, Al-Khafaji A, Davison DL, Demuth GE (2014). Validation of cell-cycle arrest biomarkers for acute kidney injury using clinical adjudication. Am J Respir Crit Care Med.

[15] Hoste EA, McCullough PA, Kashani K, Chawla LS, Joannidis M, Shaw AD, Feldkamp T, Uettwiller-Geiger DL, McCarthy P, Shi J, Walker MG, Kellum JA, Sapphire Investigators (2014). Derivation and validation of cutoffs for clinical use of cell cycle arrest biomarkers. Nephrol Dial Transplant.

[16] Boonstra A, Versluis A, Vos JF (2014). Implementing electronic health records in hospitals: a systematic literature review. BMC Health Serv Res.

[17] Flechet M, Grandas FG, Meyfroidt G (2016). Informatics in neurocritical care: new ideas for Big Data. Curr Opin Crit Care.

[18] Wilson T, Quan S, Cheema K, Zarnke K, Quinn R, de Koning L (2016). Risk prediction models for acute kidney injury following major noncardiac surgery: systematic review. Nephrol Dial Transplant.

[19] Moons KG, Altman DG, Reitsma JB, Ioannidis JP, Macaskill P, Steyerberg EW (2015). Transparent Reporting of a multivariable prediction model for Individual Prognosis or Diagnosis (TRIPOD): explanation and elaboration. Ann Intern Med.

[20] Hodgson LE, Sarnowski A, Roderick PJ, Dimitrov BD, Venn RM, Forni LG (2017). Systematic review of prognostic prediction models for acute kidney injury (AKI) in general hospital populations. BMJ Open.

[21] Hanley JA, McNeil BJ (1982). The meaning and use of the area under a receiver operating characteristic (ROC) curve. Radiology.

[22] Cook NR (2007). Use and misuse of the receiver operating characteristic curve in risk prediction. Circulation.

[23] Hosmer DW, Lemeshow S (2000). Applied logistic regression.

[24] Fitzgerald M, Saville BR, Lewis RJ (2015). Decision curve analysis. JAMA.

[25] Malhotra R, Kashani KB, Macedo E, Kim J, Bouchard J, Wynn S (2017). A risk prediction score for acute kidney injury in the intensive care unit. Nephrol Dial Transplant.

[26] Flechet M, Güiza F, Schetz M, Wouters P, Vanhorebeek I, Derese I (2017). AKIpredictor, an online prognostic calculator for acute kidney injury in adult critically ill patients: development, validation and comparison to serum neutrophil gelatinase-associated lipocalin. Intensive Care Med.

[27] Casaer MP, Mesotten D, Hermans G, Wouters PJ, Schetz M, Meyfroidt G (2011). Early versus late parenteral nutrition in critically ill adults. N Engl J Med.

[28] Deng Y, Chi R, Chen S, Ye H, Yuan J, Wang L (2017). Evaluation of clinically available renal biomarkers in critically ill adults: a prospective multicenter observational study. Crit Care.

[29] Chiofolo C, Chbat N, Ghosh E, Eshelman L, Kashani K (2019). Automated continuous acute kidney injury prediction and surveillance: a random forest model. Mayo Clin Proc.

[30] Zimmerman LP, Reyfman PA, Smith AD, Zeng Z, Zho A, Sanchez-Pinto LN (2019). Early prediction of acute kidney injury following ICU admission using a multivariate panel of physiological measurements. BMC Med Inform Decis Mak.

[31] Macedo E, Malhotra R, Claure-Del Granado R, Fedullo P, Mehta RL (2011). Defining urine output criterion for acute kidney injury in critically ill patients. Nephrol Dial Transplant.

[32] De Rosa S, Samoni S, Ronco C (2016). Creatinine-based definitions: from baseline creatinine to serum creatinine adjustment in intensive care. Crit Care.

[33] Sutherland SM, Chawla LS, Kane-Gill SL, Hsu RK, Kramer AA, Goldstein SL, Kellum JA, Ronco C, Bagshaw SM, 15 ADQI Consensus Group (2016). Utilizing electronic health records to predict acute kidney injury risk and outcomes: workgroup statements from the 15(th) ADQI Consensus Conference. Can J Kidney Health Dis.

[34] Steyerberg EW, Harrell FE Jr, Borsboom GJ, Eijkemans MJ, Vergouwe Y, Habbema JD (2001). Internal validation of predictive models: efficiency of some procedures for logistic regression analysis. J Clin Epidemiol.

[35] Steyerberg EW, Bleeker SE, Moll HA, Grobbee DE, Moons KG (2003). Internal and external validation of predictive models: a simulation study of bias and precision in small samples. J Clin Epidemiol.

[36] Kramer AA, Zimmerman JE (2007). Assessing the calibration of mortality benchmarks in critical care: The Hosmer-Lemeshow test revisited. Crit Care Med.

[37] Hodgson LE, Sarnowski A, Roderick PJ, Dimitrov BD, Venn RM, Forni LG (2017). Systematic review of prognostic prediction models for acute kidney injury (AKI) in general hospital populations. BMJ Open.

[38] Caragata R, Wyssusek KH, Kruger P (2016). Acute kidney injury following liver transplantation: a systematic review of published predictive models. Anaesth Intensive Care.

[39] Huen SC, Parikh CR (2012). Predicting acute kidney injury after cardiac surgery: a systematic review. Ann Thorac Surg.

[40] Moher D, Liberati A, Tetzlaff J, Altman DG, PRISMA Group (2009). Preferred Reporting Items for Systematic Reviews and Meta-Analyses: the PRISMA statement. Ann Intern Med.

